# Infliximab-associated fulminant hepatic failure in ulcerative colitis: a case report

**DOI:** 10.1186/s13256-015-0730-5

**Published:** 2015-10-30

**Authors:** Rogerio Serafim Parra, Marley Ribeiro Feitosa, Vanessa Foresto Machado, Leandra Naira Zambelli Ramalho, Jose Joaquim Ribeiro da Rocha, Omar Feres

**Affiliations:** 1 Division of Coloproctology, Department of Surgery and Anatomy, Medical School of Ribeirão Preto, University of São Paulo, Avenida dos Bandeirantes, 3900, Ribeirão Preto, SP Zip: 14048-900 Brazil; 2 Department of Pathology, Medical School of Ribeirão Preto, University of São Paulo, Avenida dos Bandeirantes, 3900, Ribeirão Preto, SP Zip: 14048-900 Brazil

**Keywords:** Hepatitis, Infliximab, Liver failure, Liver transplantation, Ulcerative colitis

## Abstract

**Introduction:**

Infliximab, an antibody against tumor necrosis factor alpha, is used to treat inflammatory bowel disease and has well-established efficacy and proven safety. Complications of this treatment are related to immunosuppression and include higher risk of serious infections and malignant neoplasia. Although extremely rare, fulminant liver damage related to infliximab therapy has been reported.

**Case presentation:**

We present the case of a 38-year-old Afro-Brazilian woman with refractory ulcerative colitis who was started on infliximab. She had no previous history of liver disease, alcohol abuse, or infection. After the fifth dose of the medication, drug-induced liver injury was diagnosed. Treatment was discontinued but our patient’s condition was aggravated by severe cholestasis and grade III/IV encephalopathy, requiring liver transplantation.

**Conclusion:**

Drug-induced liver injury is an uncommon complication of infliximab. Current consensus recommends screening for liver dysfunction prior to and during therapy. This case emphasizes the need for vigilance and highlights a rare and potentially lethal complication.

## Introduction

Antibodies against tumor necrosis factor alpha, such as infliximab (IFX), are increasingly being used to treat inflammatory bowel disease [1, 2]. They were originally used to manage moderate to severe active Crohn’s disease but have recently been approved for severe ulcerative colitis (UC) [3]. The most worrisome adverse effects of IFX are the increased risks of developing serious infections and malignancies; therefore, close monitoring of patients is required to increase safety. Although uncommon, IFX therapy has been associated with drug-induced liver injury, which frequently occurs 13 weeks after its initiation but may be diagnosed up to 6 months later [4–11]. We report a rare case of severe UC treated with IFX that was complicated by fulminant hepatic failure and required urgent liver transplantation.

## Case presentation

**Fig. 1:**
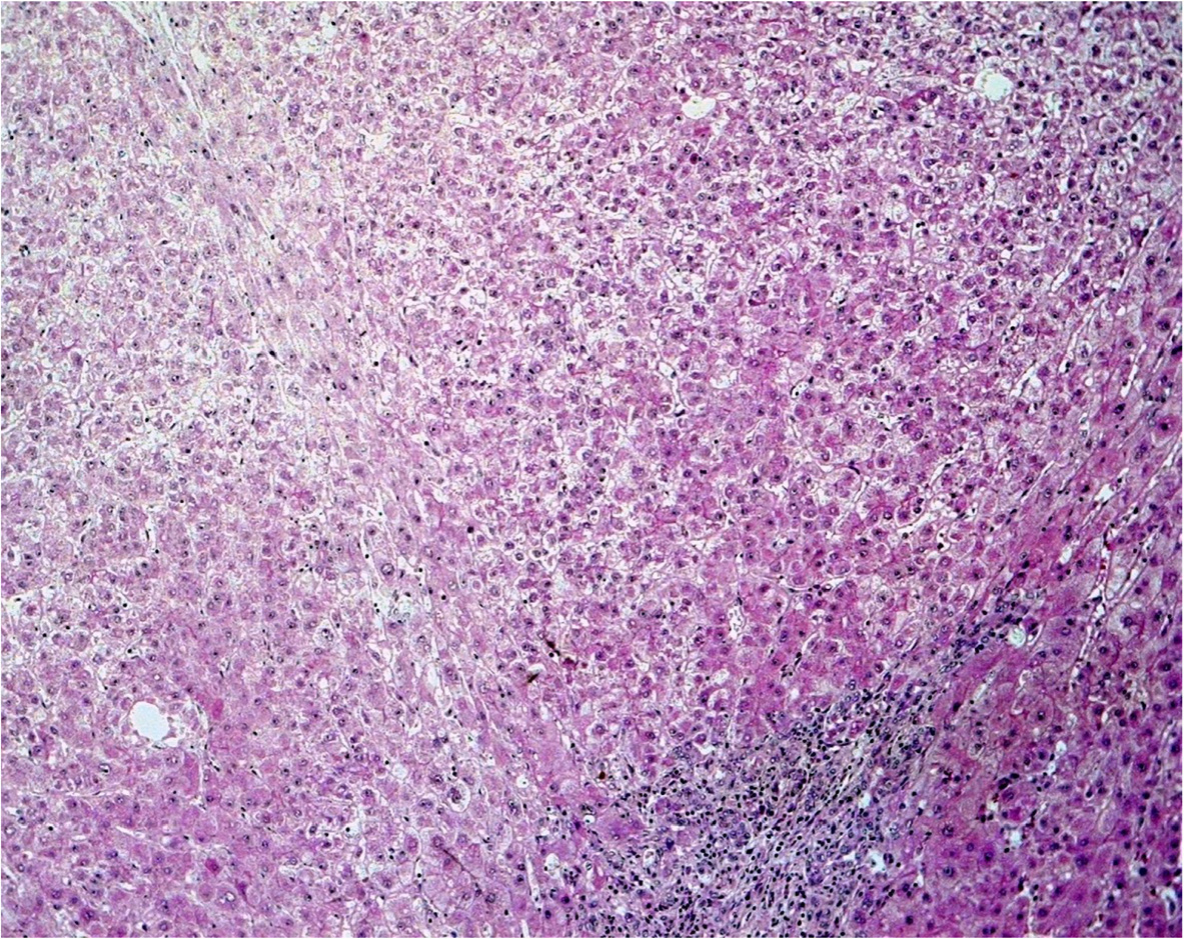
Hepatic necrosis. Hematoxylin and eosin staining, original magnification 10×

**Fig. 2:**
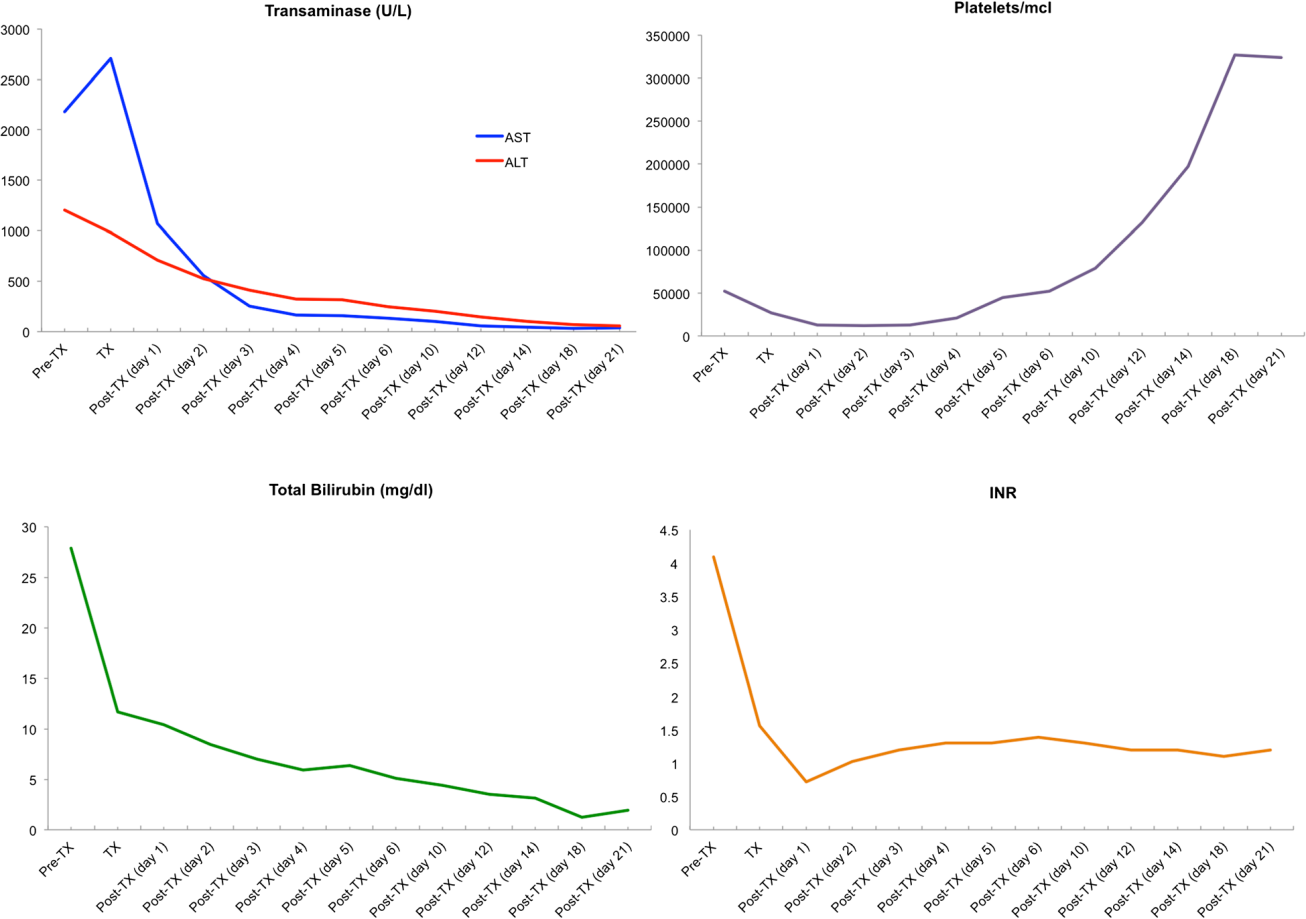
Laboratory examinations before (Pre-TX) and after (Post-TX) the liver transplant, expressed in days. Shown are transaminase levels (aspartate aminotransferase [*ALT*], shown in *blue*; alanine aminotransferase [*ALT*], shown in *red*), bilirubin levels, plasma internationalized normalized ratio (*INR*), and platelet count. *IFX* infliximab, *TX* transplant

A 38-year-old Afro-Brazilian woman with refractory UC was started on IFX (5 mg/kg). Her baseline liver and renal functions were entirely normal. Our patient became asymptomatic after IFX-induction therapy (weeks 0, 2, and 6). After the fifth dose of infliximab, routine laboratory examinations showed an increase in transaminases levels, with aspartate aminotransferase (AST) of 325 U/L and alanine aminotransferase (ALT) of 408 U/L, and the medication was discontinued. Our patient had no history of alcohol abuse or infection. Serology tests for hepatitis B and C viruses, human immunodeficiency virus (HIV), cytomegalovirus, and Epstein–Barr virus were negative. No serum smooth-muscle, anti-nucleic, or antimitochondrial antibodies could be detected. Even after we discontinued therapy, our patient’s condition worsened. She developed severe cholestasis with marked jaundice and fatigue, requiring hospitalization. Laboratory tests were as follows: bilirubin 23.38 mg/dL (normal range <1.2 mg/dL), AST 1,254 U/L (normal range <35 U/L), and ALT 2,177 U/L (normal range <105 U/L). Her platelet count decreased to 54,000 and the plasma internationalized normalized ratio (INR) was elevated to a value of 4.1. A nuclear magnetic resonance scan demonstrated acute liver damage. She developed grade III encephalopathy with marked confusion and incomprehensible speech and had an emergency whole liver transplantation. The surgery was successful. A histopathological analysis of her explanted liver revealed extensive necrosis compatible with fulminant hepatic failure (Fig. 1). After the procedure, results from hepatic tests normalized (Fig. 2). Our patient made a full recovery and is receiving immunosuppressive therapy with tacrolimus. No signs of liver rejection or colitis reactivation have yet been noted.

## Discussion

The number of patients treated with IFX has rapidly increased worldwide. An estimated 2 million people were exposed to this drug from 1998 to 2014. The colectomy rate in UC significantly decreased after the introduction of this class of drugs [12–15]. Despite this notable success, physicians must be aware of the possible complications, especially those related to immunosuppression such as serious infections and malignant neoplasia.

Elevated liver enzymes (especially ALT) have been reported during IFX treatment but are usually transitory and have no clinical implications. Fulminant liver failure is an extremely rare and serious event that requires a liver transplant, with high morbidity and mortality [16–18]. IFX-induced acute liver failure may be explained in three possible ways: autoimmune hepatitis, cholestatic injury, and direct toxicity [19].

In the present case, our patient had no history of alcohol consumption or concomitant use of any hepatotoxic drugs. Serological tests for infectious hepatitis, HIV, or other viruses were negative. Autoimmune disease was also excluded. Histopathological analysis of the explanted liver evidenced diffuse hepatitis intertwined with areas of necrosis, suggesting direct liver damage. A diagnosis of IFX-induced hepatitis was made considering the temporal relationship with IFX exposure, lack of other possible causes of liver injury, laboratory changes, and clinical deterioration.

Current guidelines support screening for liver dysfunction at 4-month intervals. It is also important to rule out any hepatotoxic risk factor prior to IFX therapy. Discontinuation of IFX is recommended if transaminase levels reach three times the upper normal limits, especially if associated with clinical manifestations [20].

## Conclusion

This report calls attention to a rare and potentially lethal adverse effect of IFX. All efforts should be made to rule out any pre-existing liver disease before initiating IFX therapy and vigilance must continue during the maintenance treatment, which must be interrupted if aminotransferases elevate more than three times above the normal levels. Signs of abrupt clinical deterioration should raise suspicion for fulminant liver disease.

## Consent

Written informed consent was obtained from the patient for publication of this case report and accompanying images. A copy of the written consent is available for review by the Editor-in-Chief of this journal.

## Abbreviations

AST: aspartate aminotransferase
ALT: alanine aminotransferase
HIV: human immunodeficiency virus
IFX: infliximab
INR: internationalized normalized ratio
UC: ulcerative colitis
